# The distinctive geographic patterns of common pigmentation variants at the *OCA2* gene

**DOI:** 10.1038/s41598-020-72262-6

**Published:** 2020-09-22

**Authors:** Kenneth K. Kidd, Andrew J. Pakstis, Michael P. Donnelly, Ozlem Bulbul, Lotfi Cherni, Cemal Gurkan, Longli Kang, Hui Li, Libing Yun, Peristera Paschou, Kelly A. Meiklejohn, Eva Haigh, William C. Speed

**Affiliations:** 1 Professor Emeritus, Department of Genetics, Yale University School of Medicine, P.O. Box 208005, New Haven, CT 06520-8005 USA; 2 Biological and Environmental Sciences, Troy University, Dothan, AL 36303 USA; 3 Institute of Forensic Science, Istanbul University–Cerrahpasa, Istanbul, 34500 Turkey; 4 Laboratory of Genetics, Immunology and Human Pathologies, Faculty of Sciences of Tunis, University of Tunis El Manar, 2092 Tunis, Tunisia; 5 Higher Institute of Biotechnology of Monastir, Monastir University, 5000 Monastir, Tunisia; 6 Turkish Cypriot DNA Laboratory, Committee on Missing Persons in Cyprus Turkish Cypriot Member Office, Nicosia, North Cyprus) Turkey; 7 Dr. Fazıl Küçük Faculty of Medicine, Eastern Mediterranean University, Famagusta (North Cyprus), Turkey; 8 Key Laboratory forMolecular GeneticMechanisms and Intervention Research On High Altitude Disease of Tibet Autonomous Region, School of Medicine, Xizang Minzu University, Xianyang, 712082 Shaanxi China; 9 Key Laboratory of High Altitude Environment and Genes Related To Disease of Tibet Ministry of Education, School of Medicine, Xizang Minzu University, Xianyang, 712082 Shaanxi China; 10 MOE State Key Laboratory of Contemporary Anthropology, School of Life Sciences, Fudan University, Shanghai, China; 11 Institute of Forensic Medicine, West China College of Preclinical and Forensic Medicine, Sichuan University, No.16. Section 3. RenMin Nan Road, Chengdu, 610041 Sichuan China; 12 Department of Biological Sciences, Purdue University, West Lafayette, IN USA; 13 Department of Population Health and Pathobiology, North Carolina State University, 1060 William Moore Drive, Raleigh, NC 27607 USA

**Keywords:** Population genetics, Genetic variation

## Abstract

Oculocutaneous Albinism type 2 (*OCA2*) is a gene of great interest because of genetic variation affecting normal pigmentation variation in humans. The diverse geographic patterns for variant frequencies at *OCA2* have been evident but have not been systematically investigated, especially outside of Europe. Here we examine population genetic variation in and near the *OCA2* gene from a worldwide perspective. The very different patterns of genetic variation found across world regions suggest strong selection effects may have been at work over time. For example, analyses involving the variants that affect pigmentation of the iris argue that the derived allele of the rs1800407 single nucleotide polymorphism, which produces a hypomorphic protein, may have contributed to the previously demonstrated positive selection in Europe for the enhancer variant responsible for light eye color. More study is needed on the relationships of the genetic variation at *OCA2* to variation in pigmentation in areas beyond Europe.

## Introduction

Oculocutaneous Albinism type 2 (*OCA2*) is a gene of interest for several reasons, not the least of which is its role in oculocutaneous albinism with about 30% of worldwide cases accounted for by 154 mutations in the *OCA2* gene^1^. Two amino acid substitutions in the coding sequence were shown by Sviderskaya et al.^2^ to be associated with decreased expression of the *OCA2* protein but not full ocular albinism. *OCA2* was subsequently studied for its association with eye color but common variants are associated not just with variation in eye color but also with variation in skin color^3–5^. Different polymorphisms in the regulatory and coding regions are primarily associated with different eye, hair, and skin pigmentation phenotypes and show large frequency differences among populations from different parts of the world.

Single nucleotide polymorphisms (SNPs) in the molecular region of *OCA2* were first implicated in inheritance of eye color variation in Europeans^6^. The strongest evidence was for variation upstream of the *OCA2* coding sequences in one of the introns of *HERC2*^7^, supported by broader population genetics studies^8^. Sturm et al.^7^ showed that rs12913832 disrupted a conserved regulatory region; the region was subsequently confirmed to be an enhancer of *OCA2*^9^. Functional variation in the *HERC2* coding sequences seems unrelated to eye color^7^. *OCA2* also has four commonly occurring SNPs that cause amino acid substitutions: rs1800414 (His615Arg), rs74653330 (Ala481Thr), rs1800407 (Arg419Gln), and rs1800401 (Arg305Trp). The Ala481Thr (rs74653330) and Val443Ile (rs121918166) variants were shown^2^ to be hypomorphic but not pathogenic in their studies of ocular albinism. The Val443Ile missense variant (rs121918166) has been reported at < 1% in Scandinavian populations^10^. These missense SNPs are distributed across 63 kb of the gene (Table 1); the enhancer SNP (rs12913832) is 38.6 kb from the start of the coding sequence.

**Table 1:** Five commonly occurring and one rare functional SNP at *OCA2* influencing expression of human pigmentation variation.

Nucleotide position GRCh38	Distance to next SNP in basepairs	dbSNP rs-number	Ancestral amino acids^‡^ Anc-position#-Drv	Alleles Anc, Drv forward strand	Hypomorphic
27,951,891	31,516	rs1800414	His615Arg	T,C	
27,983,407	1,694	rs74653330	Ala481Thr	C,T	mildly
27,985,101	71	rs121918166 *	Val443Ile	C,T	very
27,985,172	29,735	rs1800407	Arg419Gln	C,T	possibly
28,014,907	66,967	rs1800401 *	Arg305Trp	G,A	
28,081,874	38,598	–	initiation codon	–	
28,120,472		rs12913832	enhancer	A,G	

*SNPs not studied in this report.

^‡^Anc, ancestral; Drv, derived; amino acids–Ala, alanine; Arg, arginine; Gln, glutamine; His, histidine; Ile, isoleucine; Thr, threonine; Trp, tryptophan; Val, valine.

Three of the *OCA2* missense SNPs (rs1800414, rs74653330, rs1800407) have been studied in conjunction with pigmentation phenotypes, primarily in European and East Asian populations where the variants are most common. Walsh et al.^11–13^ found that including the genotype at rs1800407 in a regression equation improved the ability to predict eye color in their samples. Edwards et al.^14^ and Yuasa et al.^15–17^ found that rs1800414 was associated with skin color variation among individuals of East Asian ancestry. Eaton et al.^18^ studied both rs1800414 and rs74653330 on East Asians and found them to be independently associated with skin color. Rawofi et al.^19^ confirmed the association of rs1800414 with skin color and found it significantly associated with iris color. Lee et al.^20^ identified the derived allele at rs74653330 at a frequency of about 1% in Europeans. This hypomorphic OCA2*481Thr (rs74653330) allele was later found to be moderately frequent in many East Asian populations^17,21^.

Evidence of recent selection for the derived allele of rs12913832 at the enhancer is clearly documented in European populations as is selection for the derived allele at rs1800414 in East Asia^8^. The skin color effects of rs1800414 have been considered an example of parallel evolution for light skin color^14^. We are interested in these and other aspects of the population genetics of the *OCA2* variants. To that end we have tested (Table 1) four of the functional SNPs in the large number of population samples we have available^22^. We have also retrieved data on these SNPs from the 1,000 Genomes (1 KG) project website^23^ in those populations and assembled the published data on population frequencies. The derived alleles show very distinct biogeographic variation. That global pattern of variation is the focus of this paper.

## Methods

### Markers and Populations

Table 1 lists the three amino acid substitution SNPs at *OCA2*, rs1800414, rs74653330, and rs1800407 and the *OCA2* enhancer SNP, rs12913832, in an intron of *HERC2*, that are the focus of this study. Data on all four of these SNPs come primarily from our genotyping studies (76 populations), from a collaboration with co-author Longli Kang (7 populations), and from the 22 relatively unadmixed populations of the 1 KG project (Phase 3)^23^. Additional individual SNP frequencies were obtained primarily from the published literature and were entered into the ALFRED database (https://alfred.med.yale.edu) before it became static. A fourth amino acid substitution, rs1800401 (Arg305Trp), has been typed in the 1 KG samples but is not included here because it has been otherwise studied largely in samples defined by pigmentation phenotypes (eye, hair, skin color) in a few populations^3,24,25^. The rare amino acid change (Val443Ile) at SNP rs121918166 has only been studied on a small number of European populations^10^ and studied for its effect on eye, hair, and skin color. Only three of the 1 KG populations, all European, have the variant allele at rare frequencies ranging from 0.5% to 0.9%. All of the samples were collected with informed consent for population genetic studies such as this. Because all samples are completely anonymous, the allele frequency collection in this study is not considered human research.

### Marker Typing

Various methods were used to type the SNPs and are described in the multiple sources of the data. The source of data for each population sample is listed in Table S1 of supplemental data. The populations typed in Kidd Lab as part of this study were typed using TaqMan SNP Genotyping Assays obtained from Applied Biosystems as previously described; data on some of the SNPs in some of the populations were previously published^8,26^.

### Statistics

As these SNPs are simple co-dominant genetic systems allele frequencies were estimated by simple gene counting. The density plots were produced by Surfer (version 12.8) software (https://www.goldensoftware.com). The haplotype frequencies were estimated using Phase version 2.1.1^27,28^. Each population was phased separately.

## Results and discussion

We have assembled data on 238 population samples with allele or genotype frequencies for at least one of the four commonly studied variants. Most of those studies have data on two or more of the SNPs (Table S1). 105 population samples have data for all four of those SNPs at *OCA2*: three amino acid substitution SNPs at *OCA2*, rs1800414, rs74653330, and rs1800407 and the *OCA2* enhancer SNP, rs12913832, in an intron of *HERC2*. The population samples with *OCA2* data are listed in Supplemental Table S1.

### Individual SNP frequencies

**Figure 1:**
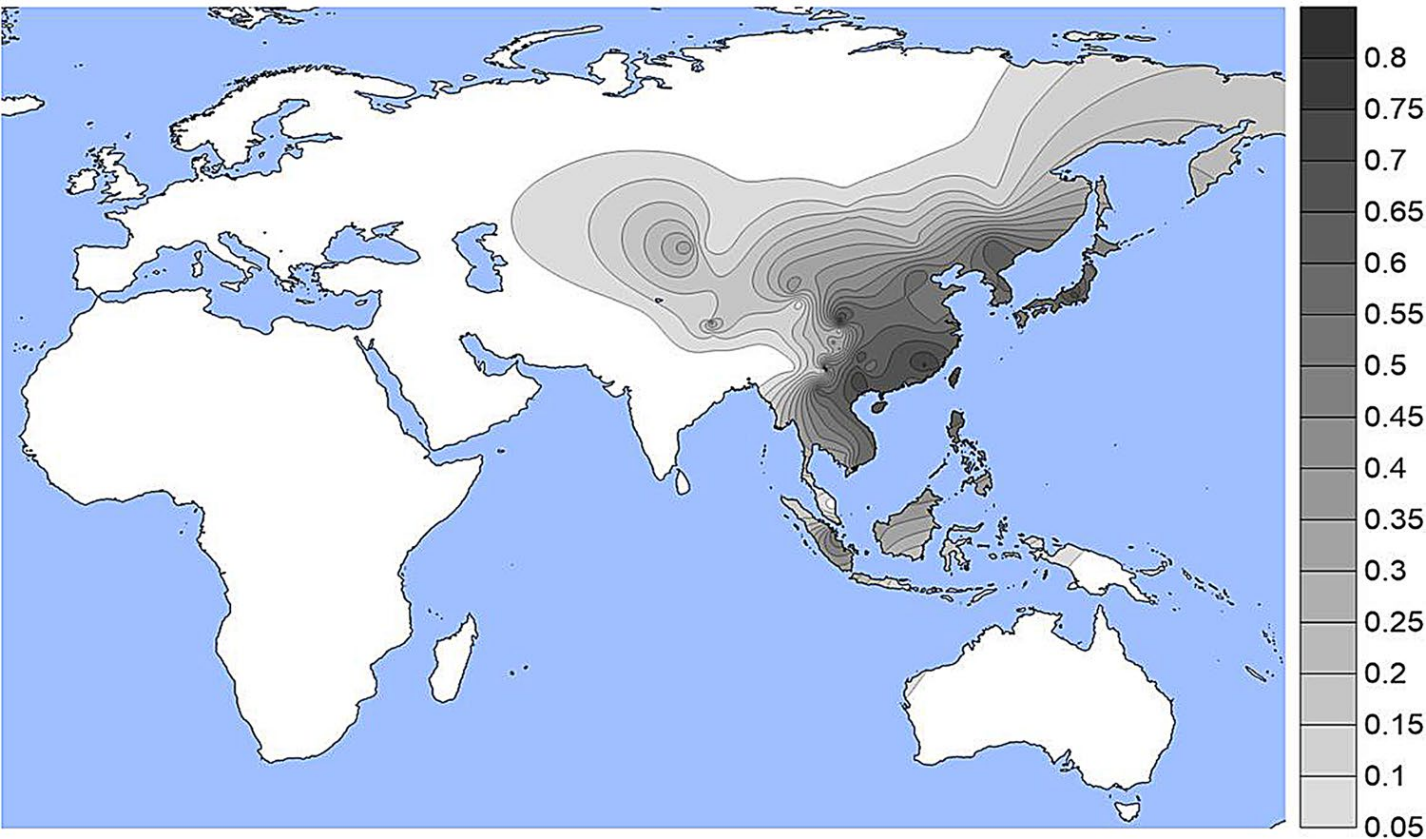
A density plot of the frequencies of the derived allele at rs1800414. The underlying data for Figs. 1, 2, 3 and 4 are in Table S1. Alternative graphic representation with the frequencies of each population sample is in Fig. S1. See text.

**Figure 2:**
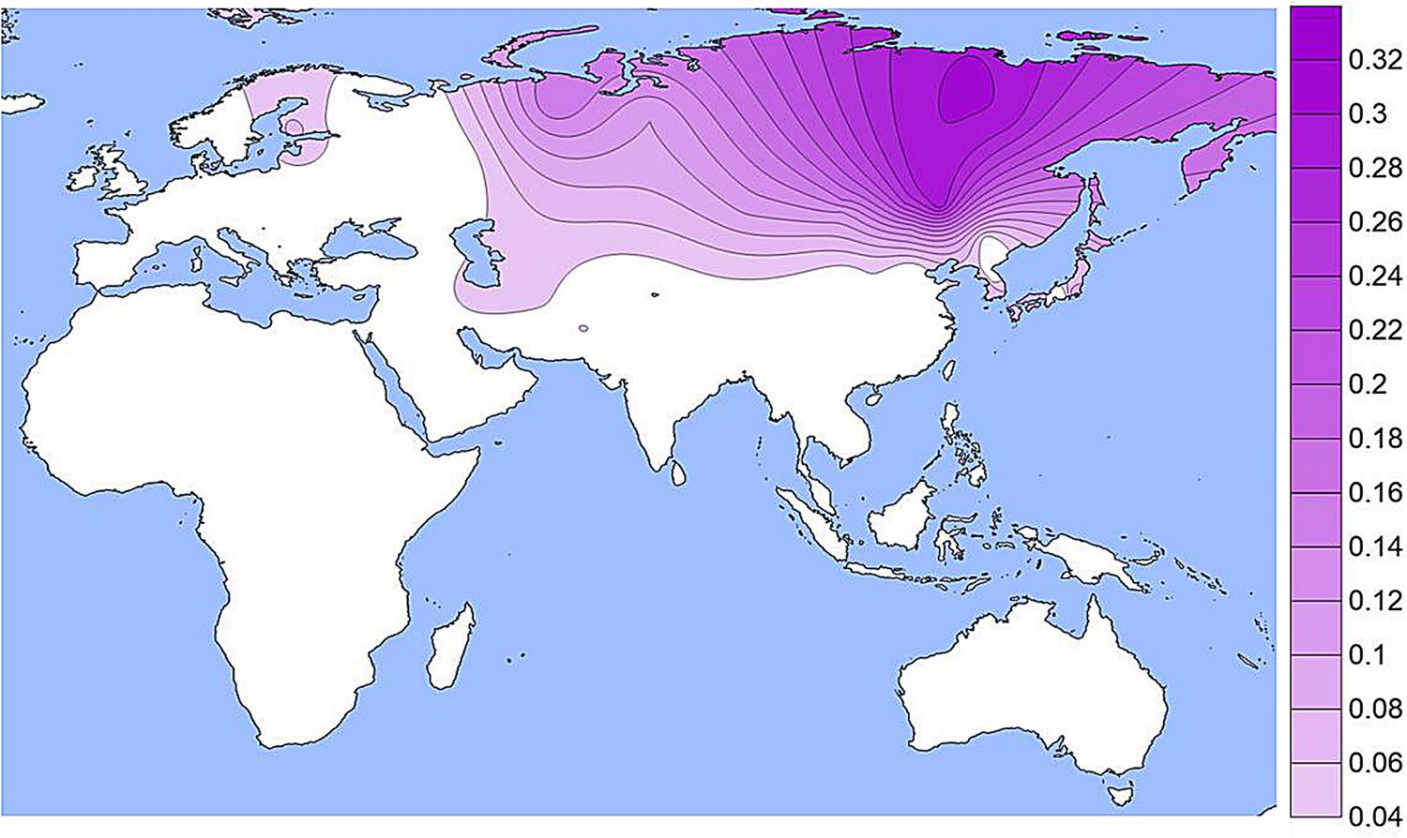
A density plot of the frequencies of the derived allele at rs74653330. The scale has been adjusted to minimize visual extrapolation to very rare occurrences. An outlier frequency of 0.52 in a small Orogen sample was omitted from the density plot and the omission resulted is a slight shift of the highest frequency region to the West. See Figure S2, caption for Fig. 1, and text.

**Figure 3:**
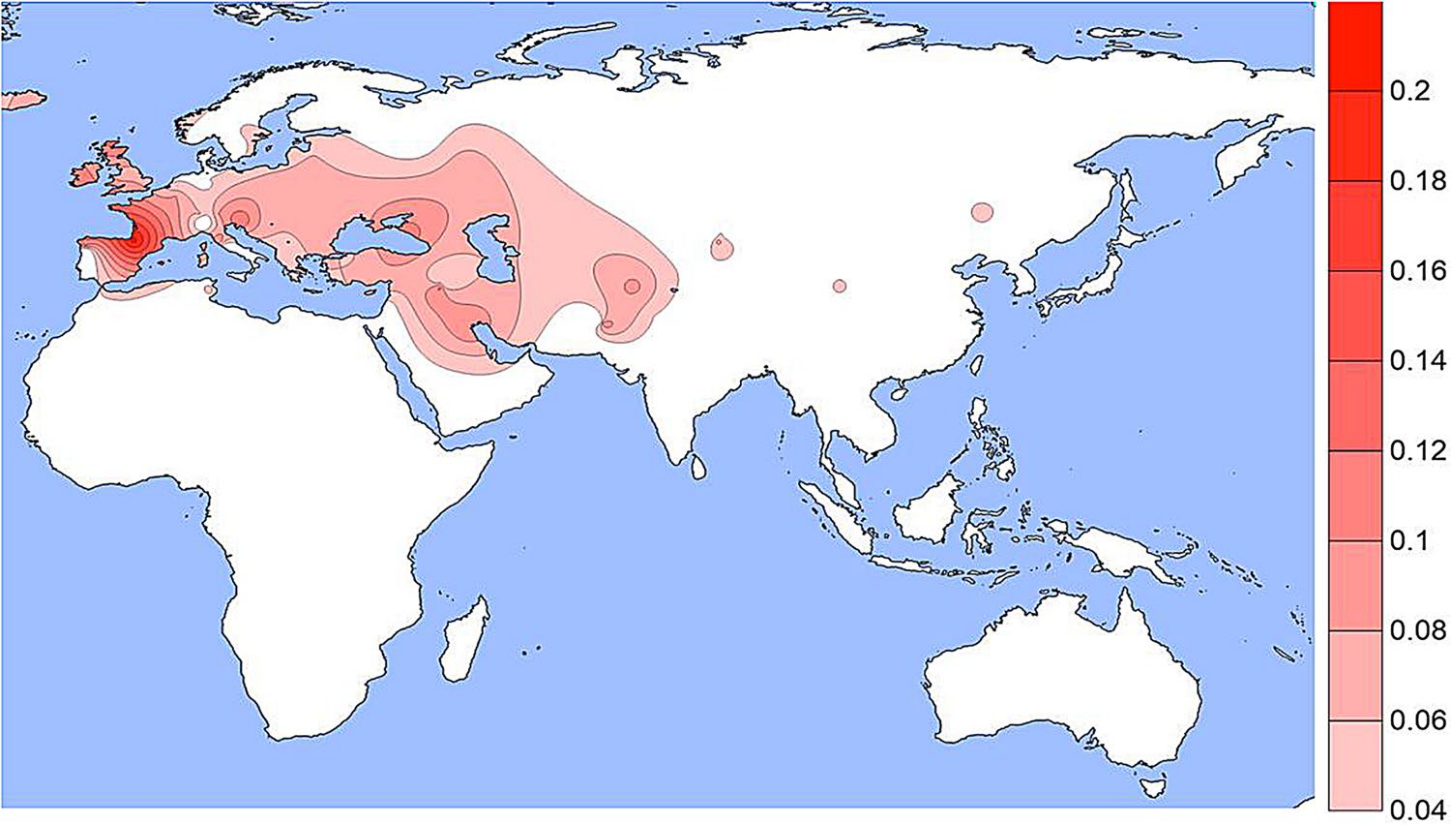
A density plot of the frequencies of the derived allele at rs1800407. The scale has been adjusted to minimize visual extrapolation to very rare occurrences. See Fig. S3, caption for Fig. 1, and text.

**Figure 4:**
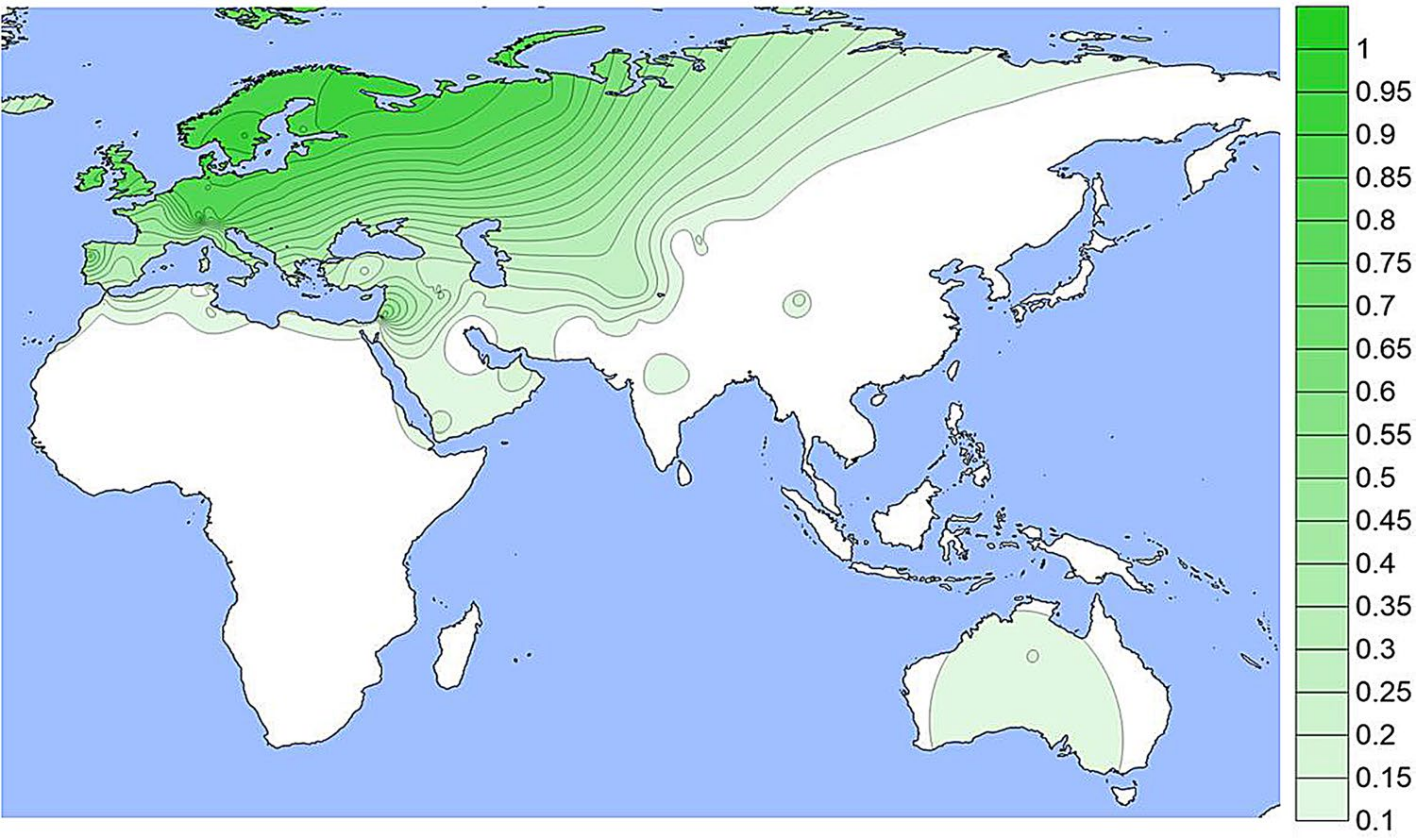
A density plot of the frequencies of the derived allele at rs12913832. The scale has been adjusted to minimize visual extrapolation to very rare occurrences. See Fig. S4, caption for Fig. 1, and text.

The population specific allele frequencies of the four functional SNPs noted in Table 1 are given in Supplemental Table S1 and presented as density plots in Figs. 1, 2, 3 and 4; a different graphic representation indicating the frequency data for each specific population sample is given in Supplemental Figs. S1 through S4. All of the functional SNPs have data for many population samples. Each of the Supplemental figures includes all of the population samples with data for any of the SNPs; blanks represent missing data for a given population sample. Each bar in the Supplemental figures represents the data from a single population study involving that SNP; there are several instances of multiple independent samples for the same ethnic/geographic group.

The derived allele at rs1800414 is largely restricted to but common in many East Asian populations (Figs. 1 and S1). This SNP has been studied in many populations that have not been studied for various of the other three SNPs. This variant reaches frequencies over 50% in most of East and Southeast Asia. It has lower frequencies of 5% to 15% in the Pacific populations and in Central and Northern Asia as well as Tibet and other parts of Southwestern China.

The derived allele at the missense SNP, rs74653330 (Ala481Thr) (Figs. 2 and S2) has been studied less comprehensively than rs1800414 but occurs widely in Northern Eurasia and is especially common in Eastern Siberian and Mongolian populations The report of a frequency of 52% in the Oroqen (sampled in northern China near the Russian border) is an outlier in terms of frequency but not geography: it was omitted from Fig. 2 but not Fig. S2. Off the scale of Fig. 2 (frequencies < 4%) the derived allele occurs rarely in most of Europe, in some Southwest Asian populations (Turkish, Iranians), in South Asia (Hazara), and in China (Tibetans). In northern Europe it occurs at low frequencies (1% to 3%) in some populations (Chuvash, Vologda Russians) and reaches 5% to 7% in Finnish samples. Given that the derived allele at rs74653330 is hypomorphic, it is a clear candidate for studies of selection favoring the allele in the northern populations.

The derived allele at rs1800407 (Figs. 3 and S3) occurs at low frequencies in most populations in North Africa, Europe, South Asia, and in some populations in East Asia but mostly off the scale in Fig. 3 which is driven primarily by a few values greater than 10% frequency. For example, in 18 Spanish Basques the frequency is 21% while in 14 Orcadians, the frequency is 14%.

The rs12913832 SNP (Figs. 4 and S4) is the enhancer polymorphism and has the largest number of population samples with data since most studies of other pigmentation SNPs have also included rs12913832. This variant is well known for high frequencies in Northern Europe (70% to 95%) as seen in Figs. 4 and S4. It is found at more moderate frequencies in populations from Southern Europe, Southwest Asia, North Africa, and at lower frequencies (5% to 20%) in South and Central Asia. It is seen less frequently in North and East Asia and in the Native American populations. While admixture of Europeans in Native American populations is common, our studies overall show very low frequencies in our specific population samples except for the Maya sample (Fig. S4). Given the evidence of the variant in Northern Asia, the likely ancestral region for Native Americans, it is possible that the existence of the promoter variant at a low frequency in Native Americans is ancestral and not due to recent admixture. The same possibility applies to the presence in Australian Aborigines. The subset of 39 less admixed Australian Aborigines have a 15% frequency compared to a frequency of 40% in the full sample of 102 Aborigines.

SNPs rs1800401 and rs121918166 have not been studied in as many populations as any of the four other SNPs and we have not considered them in this study. The variant at rs121918166 has only been observed at rare frequencies in Scandinavians. Based on the populations in the 1 KG the derived allele at rs1800401 occurs most frequently, 10% to 20%, in African and South Asian populations and is absent to < 12% in East Asia and Europe.

Evidence argues that the variant alleles at the four common SNPs depicted in Figs. 1, 2, 3 and 4 are functional^2,8,9,11,17,24^. Each of the four variants has a distinct geographic distribution but overlaps exist. In East Asia the hypomorphic rs74653330 allele overlaps somewhat with the rs1800414 variant but they appear to occur on separate haplotypes in the population. However, both the enhancer variant at rs12913832 and the amino acid substitution at rs1800407 occur frequently in Europe and surrounding areas and occur on the same chromosome at some unclear frequency.

### Two SNP haplotype–rs1800407 and rs12913832

The interaction between the rs12913832 and rs1800407 loci is interesting. The variant allele at rs1800407 has been included in the equations used for eye color prediction^11^ for nearly a decade and was suggested by Sturm et al.^7^ as functioning to increase the penetrance of the enhancer variant. Duffy et al.^29^ notes that heterozygosity for the derived allele at rs1800407 decreases the probability of green eyes on the homozygous derived rs12913832 background but increases it on a heterozygous rs12913832 background. Several studies have referred to the relationship of rs1800407, especially the 419Gln allele, and the enhancer variant as an example of epistasis^29–32^. However, if we consider the functional unit as production of a protein we necessarily include the rate of production of mRNA and the coding content of that mRNA. The term *epistasis* seems inappropriate because these two DNA variants are not functionally independent loci. The haplotype is the functional unit and the locus can be considered as a four-allele locus, at least with respect to the enhancer and rs1800407 (Table 2). The phenotypes determined by three of the alleles (haplotypes) are clear; the fourth is not clear from existing studies.

**Table 2:** Haplotypes of the ancestral 419Arg and derived 419Gln alleles at rs1800407 and the enhancer normal (E +) and negative (E-) alleles at rs12913832. The doubly-derived (cis) haplotype has frequency estimates of 1% to 3.6% in 14 European populations (see Table S2).

	2 SNP haplotype	Eye color effect
419Arg-E +	CA	Ancestral; dark iris color
419Gln-E +	TA	?
419Arg-E-	CG	Light iris color
419Gln-E-	TG	Light iris color; Doubly-derived/cis

If the doubly-derived chromosome for rs1800407 and rs12913832 results in “higher penetrance” for light eye color, the derived allele at rs1800407 must have a functional difference. While it was not studied by Sviderskaya et al.^2^, an obvious implication is that it is a hypomorphic allele. These cis chromosomes would have reduced production (because of the enhancer variant) of a hypomorphic OCA2 protein (because of the 419Gln allele at rs1800407). Selection operated on some trait to increase the frequency of the enhancer variant; this cis combination of the two variants with a presumably hypomorphic protein might have been more strongly affected.

On a background of homozygosity for the enhancer (rs12913832) variant, the frequency of heterozygotes of the amino acid substitution (rs1800407) is 246/(246 + 3,039) or 7.5% in Duffy’s largely British origin population sample. Those genotypes involve one chromosome that is doubly-derived (i.e., cis) for the two variants and one that has only the enhancer variant. On a heterozygous enhancer background genotype, however, the amino acid substitution heterozygotes occur at a higher frequency of 529/(529 + 1,248) or 29.8%. Those nearly 30% of individuals are composed of both cis and trans genotypes for the two functional variants. The evidence is consistent with those two genotypes having different phenotypes as would be predicted by considering the functional context: the cis genotype has one fully normal protein at normal amounts and one variant protein produced at reduced amounts; the trans genotype has a normal protein at reduced amounts and a variant protein at normal amounts.

The proportions of the two enhancer genotypes in that study^29^ are not necessarily in HW proportions depending on how they were ascertained, which is not specified. In fact, the ratio of the enhancer homozygotes to heterozygotes is 1.849 which is compatible with an enhancer variant frequency of about 0.79, essentially the same as in our summary (Table S1) for Northwest Europe. However, the frequency of the amino acid substitution is not so easily estimated from these data.

By maximum likelihood the phase of the ambiguous double heterozygotes will be estimated to be partly genotypes with the derived alleles in cis if there is evidence that the cis allele exists. We find (Table 3) that direct gene counting evidence of the cis haplotype is seen primarily in northern Europeans. In those populations with the gene counting evidence for this haplotype the frequency of the cis haplotype is 3%. We note the higher frequencies are in the British, Irish, and CEU samples. Several individuals in these and other populations in northern Europe and elsewhere are double heterozygotes with phase to be estimated statistically. The uncertainties of statistical phasing make it difficult with the existing sample sizes to give exact proportions of the two relevant genotypes, cis and trans. Gene counting evidence exists for both the cis and trans chromosomes; the doubly heterozygous genotype must be apportioned statistically and that is the source of uncertainty given the small numbers of the relevant genotypes setting the expectation (Table 3).

**Table 3:** Observed genotype counts for rs1800407 and rs12913832 among individuals with no missing data for these two SNPs. The groups shown are primarily the subset of 105 populations in Fig. 2 from world regions (Europe/SWAsia/NAfrica/SCAsia) where double heterozygotes were observed. The cells with bold underlined values indicate definite evidence of the cis (doubly-derived) haplotype,TG; see text.

rs1800407	CC	CT	TT	CC	CT	TT	CC	CT	TT	
rs12913832	AA	AA	AA	AG	AG	AG	GG	GG	GG	
Population										N
KSR	32	3	0	5	1	0	1	0	0	42
SOU	32	0	0	14	0	0	1	0	0	47
MHD	27	1	0	9	2	0	1	0	0	40
PLA	33	5	0	21	1	0	5	0	0	65
DRU	39	5	0	38	2	0	17	0	0	101
SRD	17	3	1	12	0	0	2	0	0	35
TCP	22	5	1	20	2	0	9	0	0	59
TRK	38	4	2	24	3	0	9	0	0	80
IRN	28	4	0	8	2	0	0	0	0	42
ADY	22	4	2	18	3	0	3	**1**	0	53
ASH	27	5	0	45	3	0	51	0	0	131
TSI	23	9	2	50	6	0	15	**2**	0	107
GRK	10	6	0	23	2	0	9	0	0	50
IBS	38	10	0	40	8	**1**	9	**1**	0	107
CHV	2	1	0	13	2	0	21	**3**	0	42
HGR	7	0	0	37	9	0	34	**2**	0	89
RUA	0	0	0	6	0	0	25	**2**	0	33
RUV	1	0	0	13	0	0	29	**1**	0	44
EAM	5	2	0	26	3	0	48	**3**	0	87
GBR	3	1	0	18	7	0	58	**3**	**1**	91
CEU	2	2	0	29	8	**1**	53	**4**	0	99
IRI	1	5	0	21	13	0	67	**7**	0	114
DAN	1	1	0	8	1	0	39	**1**	0	51
FIN	1	0	0	8	1	0	23	**1**	0	34
FN1	0	0	0	16	2	0	78	**3**	0	99
KMZ	2	0	0	15	0	0	28	0	0	45
PTH	27	1	2	6	1	0	2	0	0	39
PJL	72	7	0	14	1	0	2	0	0	96
STU	93	3	0	5	1	0	0	0	0	102
BEB	65	4	0	15	2	0	0	0	0	86
HZR	65	11	2	12	3	0	0	0	0	93
AKZ	45	2	0	13	1	0	1	0	0	62
MGL	87	1	0	10	2	0	0	0	0	100
MAY	42	1	0	4	3	0	0	0	0	50

**Figure 5:**
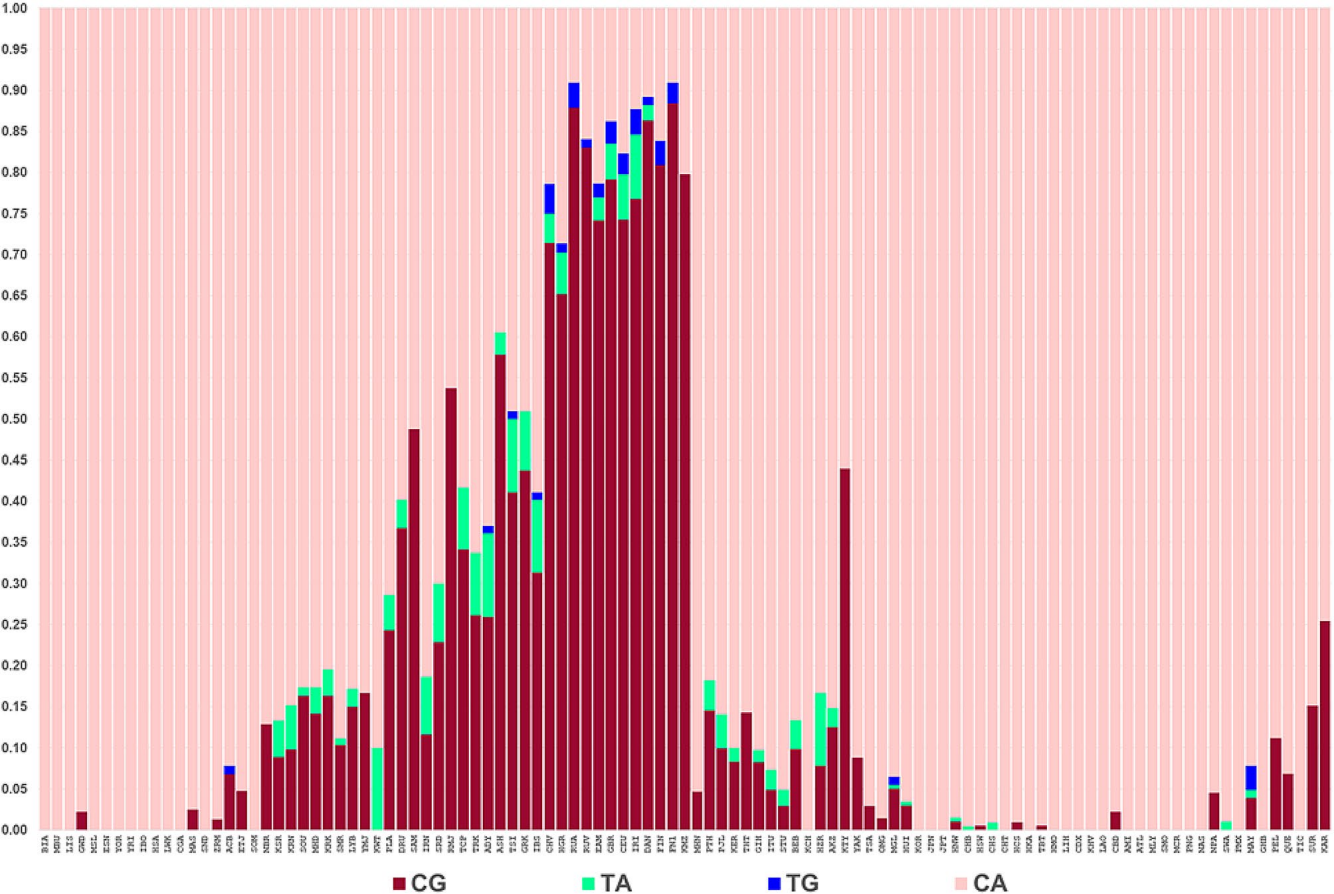
The haplotypes of rs12913832 and rs1800407 showing the high relative frequency of the doubly-derived haplotype especially in Northern Europe.

There are 10 genotypes possible for the four haplotypes of the rs12913832 and rs1800407 variants. How all of those genotypes relate to phenotypes under selection is not known. The haplotype frequency distribution bar plots of the two SNPs common in Europe, Southwest Asia, and North Africa among 105 populations are shown in Fig. 5. (The haplotype frequencies are in supplemental Table S2). In our data we have seen direct evidence for 8 of those genotypes (Table 4). The variants at rs12913832 and rs1800407 occur in cis at the highest frequencies in the northern European samples and the gene-counting evidence for the cis chromosome occurs almost exclusively in these northern European populations (Table 3). These haplotypes are relevant to how genotypes might influence pigmentation and selection in those northern European populations. While random genetic drift can always be a possible explanation for the pattern, it seems a highly unlikely explanation for the evidence of this doubly-derived chromosome to exist only in the populations for which evidence of selection on the enhancer chromosomes is strongest.

**Table 4:** Distribution of individuals (by direct gene counting and by inference) in 105 populations for the 10 possible genotypes of the 2-SNP haplotype based on rs1800407, rs12913832.

Ten possible genotypes		Direct counting	Inferred by PHASE from double heterozygotes	Inferred missing genotype	Total individuals	Observed in populations
CA	CA	4,643	0	179	4,822	
CA	CG	885	0	40	925	
CG	CG	676	0	18	694	
CA	TA	131	0	6	137	
CG	TA	0	87	0	87	
CG	TG	35	0	0	35	
TA	TA	13	0	1	14	
CA	TG	0	8	0	8	
TA	TG	2	0	0	2	CEU, IBS
TG	TG	1	0	0	1	GBR
Total		6,386	95	244	6,725	

We can expect, given the functional variation at each site, that all but the homozygote for the doubly ancestral genotype will have some positive effect toward lighter pigmentation. However, evidence for the effect on eye color of just the variant (419Gln) as heterozygous with a fully ancestral chromosome is largely absent; its expected frequency is quite small. Even assuming the haplotypes affect phenotype additively, to estimate the three different fitness parameters associated with the three derived chromosomes seems beyond the power of the existing data. We leave such estimation to others.

Many studies have reported on use of the genotypes at these sites at *OCA2* and SNPs at other genes to infer the iris, hair, and skin color of an individual from that individual’s DNA. Those efforts are most recently integrated into the HIrisplex-S web site (https://hirisplex.erasmusmc.nl/). Such phenotype inference from a DNA sample can be very useful as an investigative lead in criminal forensics. Our data summaries demonstrate that two of the SNPs, rs12913832 and rs1800414, have common variants with strikingly different geographic patterns that makes them relevant to inference of biogeographic ancestry in some parts of the world. Indeed, rs12913832, the enhancer SNP, was incorporated in the Kidd Lab panel of 55 ancestry informative SNPs^26^ and rs1800414 is part of the 74 SNPs in a panel by Li et al.^33^.

The population distribution of the chromosome with the derived enhancer variant (rs12913832) and the derived amino acid variant (419Gln for rs1800407) in cis is seen almost exclusively in northern Europe. Elsewhere, the rs1800407 variant (419Gln) occurs on a chromosome with the ancestral allele at the enhancer. The common occurrence of the doubly-derived (cis) chromosome, primarily in the populations with the strongest evidence of selection for the enhancer variant, strongly suggests selection on this chromosome in northern Europe. The north Eurasia distribution of the hypomorphic allele–481Thr at rs74653330–suggests parallel evolution for this variant as well.

Our understanding of the role of the known functional and enhancer variants in human pigmentation phenotypes has grown markedly in recent decades but, thus far, the relationships have only been studied simultaneously and in relatively large samples in a subset of populations of European and East Asian ancestry. The very strong geographical frequency patterns shown by the existing patchwork of genetic data in the *OCA2*-*HERC2* gene region are more extensive and suggest that more empirical studies are needed from more world regions so that we can refine and improve our knowledge. The studies supporting strong selection effects done thus far also support the view that more studies are important. Other genetic loci are known to influence pigmentation phenotypes. Their relative roles and the magnitude of their effects during development as well as the evolutionary impact of non-genetic factors will be more clearly understood when we have more worldwide data on the *OCA2*-*HERC2* gene region.

### Informed consent

All subjects gave permission for collection of samples and use in population studies such as this. All samples are anonymous.

## Supplementary information

Supplementary Tables.

Supplementary Figures.

## Data Availability

Allele frequencies for each of the three functional SNPs and one enhancer SNP analyzed along with the 2-SNP haplotype frequencies are in supplementary Table S1. Almost all of the individual SNP frequencies and their literature citations are also available in the ALFRED database which is freely accessible online. Five literature citations are given in Table S1 for the allele frequencies of a small number of populations that were more recently published and are not present in the static version of ALFRED.
