# Supplementary material for: The distinctive geographic patterns of common pigmentation variants at the OCA2 gene
Source: Sci Rep. 2020 Sep 22;10:15433. doi: 10.1038/s41598-020-72262-6 (PMC7508881; doi:10.1038/s41598-020-72262-6)

Supplementary Figures

accompanying manuscript
submitted to *Scientific Reports*

The distinctive geographic patterns of common pigmentation variants at the OCA2 gene

Kenneth K. Kidd [1], Andrew J. Pakstis [1], Michael P. Donnelly [1,2], Ozlem Bulbul [3], Lotfi Cherni [4,5],
Cemal Gurkan [6,7], Longli Kang [8,9], Hui Li [10], Libing Yun [11], Peristera Paschou [12],
Kelly A. Meiklejohn [13], Eva Haigh [1], William C. Speed [1]

Corresponding Author
Kenneth K. Kidd, Ph.D.
Professor Emeritus
Senior Research Scientist
Department of Genetics
Yale University School of Medicine
P.O. Box 208005
New Haven, CT 06520-8005 USA

Captions for Figures S1 to S4

Figure S1. Stacked bar representation of the allele frequencies of rs1800414. All population samples are represented in the graph; the samples with no data are blank. The 238 population samples in figures S1 through S4 are arranged in an approximate geographic order (as in Table S1) starting with Africans (mostly west to east), North Africa and Southwest Asia, Europe (roughly south to north), South Central Asia, Central Asia, Tibet, East Asia, Oceania, and the Americas (north to south).

Figure S2. Stacked bar representation of the allele frequencies of rs74653330. All population samples are represented in the graph; the samples with no data are blank. See Legend for Figure S1.

Figure S3. Stacked bar representation of the allele frequencies of rs18004407. All population samples are represented in the graph; the samples with no data are blank. See Legend for Figure S1.

Figure S4. Stacked bar representation of the allele frequencies of rs12913832. All population samples are represented in the graph; the samples with no data are blank. See Legend for Figure S1.

Figure S1. SNP rs1800414 allele frequency bar plot

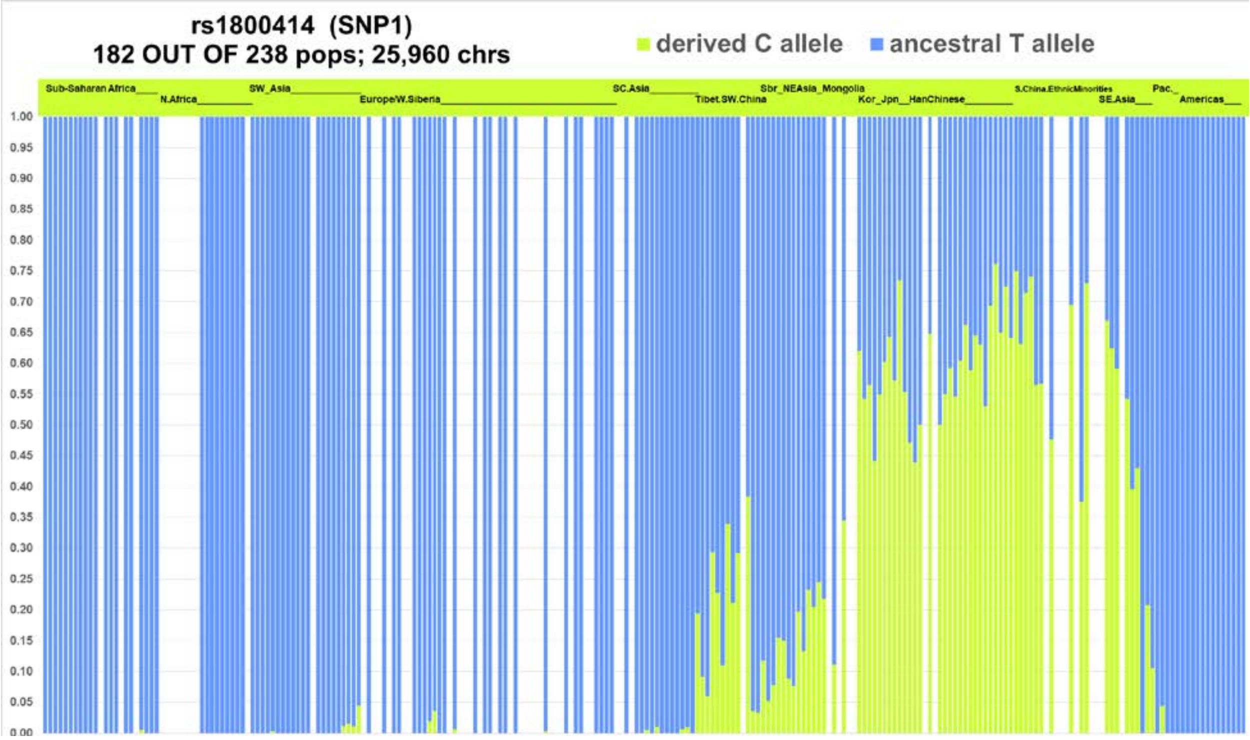

Figure S2. SNP rs74653330 allele frequency bar plot

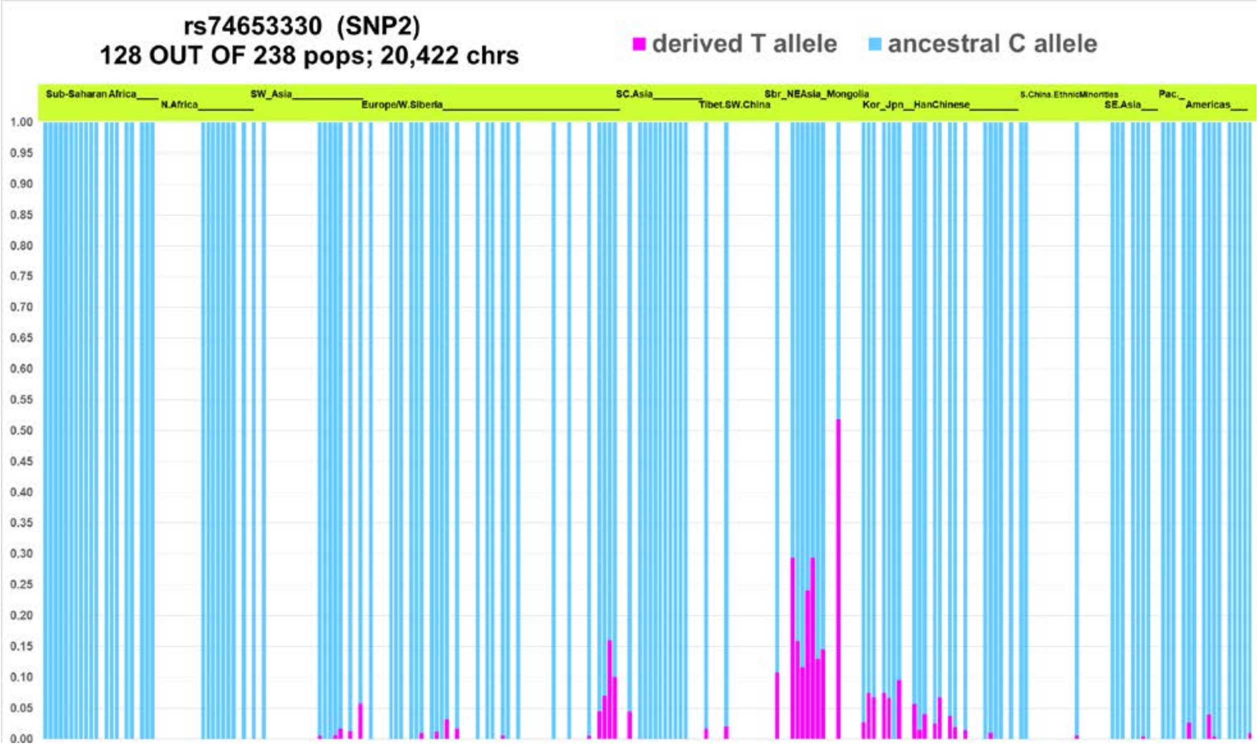

Figure S3. SNP rs1800407 allele frequency bar plot

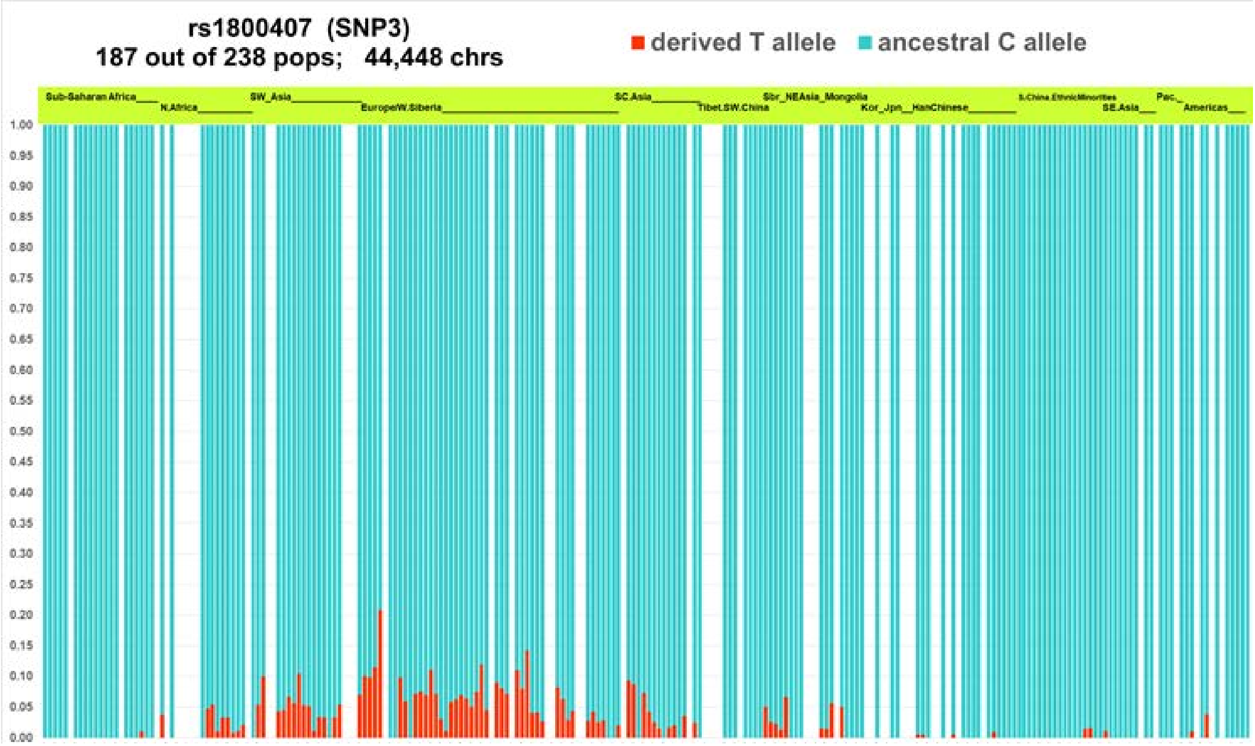

Figure S4. SNP rs12913832 allele frequency bar plot

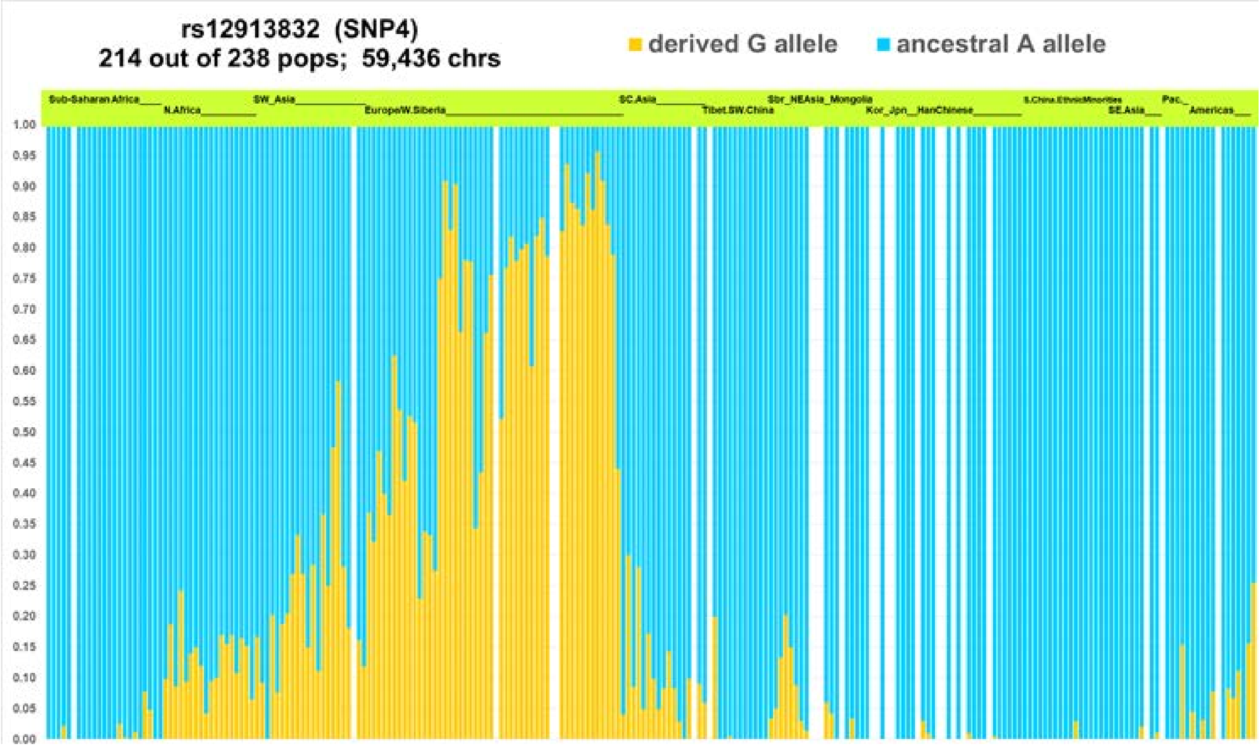

Supplement: Supplementary file 2 — Supplementary Figures. [file 41598_2020_72262_MOESM2_ESM.pdf]
